# Influence of Technological Innovations on Industrial Production: A Motif Analysis on the Multilayer Network †

**DOI:** 10.3390/e21020126

**Published:** 2019-01-30

**Authors:** Martina Formichini, Giulio Cimini, Emanuele Pugliese, Andrea Gabrielli

**Affiliations:** 1 Dipartimento di Fisica, Sapienza Università di Roma, Piazzale A. Moro 2, 00185 Rome, Italy; 2 IMT School for Advanced Studies, Piazza S. Ponziano 6, 55100 Lucca, Italy; 3 Istituto dei Sistemi Complessi (ISC), Consiglio Nazionale delle Ricerche (CNR), c/o Dipartimento di Fisica, Sapienza Università di Roma, Piazzale A. Moro 2, 00185 Rome, Italy; 4 Joint Research Centre (JRC), European Commission (EC), Edificio Expo, Calle Inca Garcilaso 3, 41092 Seville, Spain

**Keywords:** innovation system, economic complexity, multilayer networks, network motifs, maximum-entropy models, statistical validation

## Abstract

In this work we aim at identifying combinations of technological advancements that reveal the presence of local capabilities for a given industrial production. To this end, we generated a multilayer network using country-level patent and trade data, and performed motif-based analysis on this network using a statistical-validation approach derived from maximum-entropy arguments. We show that in many cases the signal far exceeds the noise, providing robust evidence of synergies between different technologies that can lead to a competitive advantage in specific markets. Our results can be highly useful for policymakers to inform industrial and innovation policies.

## 1. Introduction

Technological innovation is the main driver of modern economic growth [1,2]. It is therefore not surprising that measuring and predicting the potential impact of technological innovations on export competitiveness has been the central issue of many studies in the last forty years [3,4,5,6], as well as the focus of general interest in the field of innovation systems [7]. Several empirical studies that tried to measure such effects of national innovativeness on productivity and trade were also carried out, with mixed results [8,9]. Overall, such academic efforts provided a theoretical framework and empirical stylized facts that helped to understand the aggregate effect of innovation in determining the competitive advantage of countries in different markets. Policymakers are, however, more interested in identifying specific technologies that are relevant for specific markets [10,11], a task that is much harder to deal with in an organic and objective fashion. Indeed, the scientific effort addressing the impact of specific technologies on specific markets has been limited to ad hoc case studies that are difficult to compare [12,13].

A recent paper of ours [14] deals with this issue using a multilayer-network characterization of the innovation system. Specifically, a three-layered network of innovation activities is derived, starting from three bipartite networks describing the scientific, technological, and production activities of countries; the connections of the multilayer network represent the conditional probability that the information produced by an innovation activity (e.g., a technological sector) is used in another innovation activity (e.g., an industrial-product category) after a given time. Grounded on previous fundamental studies of economic complexity [15,16,17,18], this is the first attempt to build a representation of the innovation system as a complex multilayer network.

In this paper, we generalize this approach by measuring the potential influence that a pair of activities has on another activity. That is, we go beyond single-link analysis and consider the motifs of the multilayer network [19,20]. For simplicity, we limited our analysis to the relationships between (pairs of) technologies and products, which however represent a crucial aspect of the innovation system since the interaction between different technologies is often a driver of innovation and progress [21]. As in Reference [14], we carry out statistical validation of our results against null network models derived from maximum-entropy principles.

## 2. Materials and Methods

In order to build the bilayered network of technologies and products that is used in our analysis, we started from the following popular databases.

PATSTAT (www.epo.org/searching-for-patents/business/patstat) collects all patents from different Patent Offices around the world. The basic unit of observation in the dataset is a patent family (i.e., the set of patents with common priorities, that is, referred to the same innovation). Each family is related to the countries of origin of the applicants, and to a (set of) technological code(s) defined by the International Patent Classification (IPC). We define $W_{ct}\left(y\right)$ as the number of patent families associated to IPC code *t* applied by firms located in country *c* on year *y*.

BACI export data, recorded by UN COMTRADE (https://comtrade.un.org/), collects the import–export flows (quantified in thousands of current U.S. dollars) between countries in the world, related to production as classified using the Harmonized System 2007 of the World Customs Organization. We define $W_{cp}\left(y\right)$ as the monetary value of the overall export of country *c* for product *p* during year *y*. Note that we use export data as proxies of (competitive) industrial production, as typically done in the economic complexity literature.

In this work, we consider a data timespan ranging from 1995 to 2012, for which we have a reliable coverage for both patent and export data. For technologies, we use a 4-digit resolution of IPC codes, resulting in a number of technological sectors $N_{t}$ ranging between 629 and 636. For products, we again use a 4-digit resolution of the harmonized system, resulting in a number of product categories $N_{p}$ ranging between 1140 and 1176. Finally, the number of considered countries $N_{c}$ varies between 66 and 72. The slight variations of these numbers depend on the particular year considered, and are due to geopolitical changes and periodical recategorization of technologies and products.

Using these basic data, we can define the Revealed Comparative Advantage (RCA) [22] of a country *c* on an activity *a* (which is either a technological sector *t* or a product category *p*) in a given year *y*:

(1) $${\mathrm{RCA}}_{ca}=\frac{W_{ca}\left(y\right)}{\sum_{a^{\prime }=1}^{N_{a}}W_{ca^{\prime }}\left(y\right)}/\frac{\sum_{c^{\prime }=1}^{N_{c}}W_{c^{\prime }a}\left(y\right)}{\sum_{c^{\prime }=1}^{N_{c}}\sum_{a^{\prime }=1}^{N_{a}}W_{c^{\prime }a^{\prime }}\left(y\right)}$$

Thanks to the RCA, we can further define, for each year *y*, the binary bipartite networks countries–technologies and countries–products. These are, respectively, represented by binary biadjacency matrices $M^{C,T}\left(y\right)$ and $M^{C,P}\left(y\right)$ whose elements $M_{ct}\left(y\right)$ and $M_{cp}\left(y\right)$ are:

(2) $$M_{ca}\left(y\right)=\left\{\begin{array}{cc} 1 & {\mathrm{if}\;\mathrm{RCA}}_{ca}\left(y\right)\geq 1\\ 0 & \mathrm{otherwise} \end{array}\right.$$

(where, again, *a* refers to technological sector *t* in the case of the bipartite countries–technologies network, and to product category *p* in the countries–product case).

Once we have matrices $M^{C,T}\left(y_{1}\right)$ for year $y_{1}$ and $M^{C,P}\left(y_{2}\right)$ for the year $y_{2}$, in analogy with References [14,18], we can construct the assist matrix $B^{T\to P}\left(y_{1},y_{2}\right)$, whose generic element is defined as:

(3) $$B_{tp}\left(y_{1},y_{2}\right)=\sum_{c=1}^{N_{c}}\frac{M_{ct}\left(y_{1}\right)}{k_{t}\left(y_{1}\right)}\frac{M_{cp}\left(y_{2}\right)}{k_{c}^{\left(p\right)}\left(y_{2}\right)}\;.$$

In the above expression, $k_{t}\left(y_{1}\right)=\sum_{c=1}^{N_{c}}M_{ct}\left(y_{1}\right)$ is the number of countries having technology *t* in their technological portfolio at year $y_{1}$, and $k_{c}^{\left(p\right)}\left(y_{2}\right)=\sum_{p=1}^{N_{p}}M_{cp}\left(y_{2}\right)$ is the cardinality of the product basket of country *c* in year $y_{2}$. As explained in Reference [14], $B_{tp}\left(y_{1},y_{2}\right)$ with $y_{1}\leq y_{2}$ give the conditional probability that a bit of information produced in technological sector *t* in year $y_{1}$ arrives (via a random walk on the coupled bipartite network) at product category *p* in year $y_{2}$, through one of the countries having *t* in its technological basket at $y_{1}$ as well as *p* in its product basket at $y_{2}$. Elements $B_{tp}\left(y_{1},y_{2}\right)$ then represent the weighted links of the bilayered (or bipartite) network technologies–products, with the former at year $y_{1}$ and the latter at year $y_{2}$. These links are extensively studied in Reference [14].

Here, we move forward and consider the Λ motifs of the bipartite technologies–products network:

(4) $$\Lambda_{tt^{\prime }}^{p}\left(y_{1},y_{2}\right)=B_{tp}\left(y_{1},y_{2}\right)B_{t^{\prime }p}\left(y_{1},y_{2}\right).$$

$\Lambda_{tt^{\prime }}^{p}\left(y_{1},y_{2}\right)$ gives the conditional probability that two bits of information originally located on technologies *t* and $t^{\prime }$, respectively, at year $y_{1}$ both reach product *p* at year $y_{2}$. In other words, this motif quantifies the joint probability for co-occurrence in a single country of pair technology *t* and product *p*, and of pair technology $t^{\prime }$ and product *p*, where the two events are considered as independent. Note that, while this interpretation of the Λ motifs cannot be directly related to “impact” or “causality”, it does go beyond a simpler measure of a (time-dependent) correlation. Note also that the name Λ motif comes from the fact that, in the bipartite technologies–products network, this quantity gives the weight of a Λ-shaped set of two links having different origins in the technology layer and the same end in the product layer [20]. In principle, it is possible to generalize this approach by considering higher-order motifs by, e.g., assessing the influence of a wider group of technologies on a single product. For the sake of simplicity and due to limits of statistical significance, here we focus on the simple Λ motif.

After obtaining the empirical values of the Λ motifs from the data, we statistically validate them using their probability distribution derived from an appropriate null model, which is schematically defined as follows (see the Appendix A for a thorough presentation). For each year *y*, we build two statistical ensembles of biadjacency matrices ${\tilde{M}}^{C,T}\left(y\right)$ and ${\tilde{M}}^{C,P}\left(y\right)$, respectively, for the countries–technologies and countries–products bipartite networks. These networks are built to be maximally random, apart from having the ensemble average of node degrees equal to the observed values in the empirical networks. For node degrees, we mean both technological diversification of countries ${\tilde{k}}_{c}^{\left(t\right)}\left(y\right)=\sum_{t}{\tilde{M}}_{ct}^{y}$ and technology ubiquities ${\tilde{k}}_{t}\left(y\right)=\sum_{c}{\tilde{M}}_{ct}^{y}$ for the countries–technologies network, and analogously both product diversification of countries ${\tilde{k}}_{c}^{\left(p\right)}\left(y\right)=\sum_{p}{\tilde{M}}_{cp}^{y}$ and product ubiquities ${\tilde{k}}_{p}\left(y\right)=\sum_{c}{\tilde{M}}_{cp}^{y}$ for the countries–products network. We chose these quantities as constraints as we wanted our null model to only bear the information contained in the diversification of countries and the ubiquity of activities without taking into account the specific pattern of co-occurrences found in the empirical networks. In the spirit of information-theory interpretation of statistical mechanics [23,24,25], the probability measure defining both statistical ensembles of binary bipartite networks is obtained using a constrained entropy maximization approach. The resulting ensembles are known in the literature as Bipartite Configuration Models (BiCM) [20]. Finally, given BiCM ensembles for bipartite networks ${\tilde{M}}^{C,T}\left(y_{1}\right)$ and ${\tilde{M}}^{C,P}\left(y_{2}\right)$, we can use Equations (3) and (4) appropriately applied to BiCM quantities to derive the probability distribution for value ${\tilde{\Lambda }}_{tt^{\prime }}^{p}\left(y_{1},y_{2}\right)$ in the null model [26,27]. Numerically, we populate the BiCM ensembles by generating $10^{3}$ matrices ${\tilde{M}}^{C,T}\left(y_{1}\right)$ and ${\tilde{M}}^{C,P}\left(y_{2}\right)$, and then contract each pair to generate a final ensemble of $10^{3}$ null matrices ${\tilde{B}}^{T\to P}\left(y_{1},y_{2}\right)$.

In the following, we focus on $\Lambda_{tt^{\prime }}^{p}\left(\Delta y\right)$, that is, the average value of $\Lambda_{tt^{\prime }}^{p}\left(y_{1},y_{2}\right)$ over all pairs of years giving the same difference $y_{2}-y_{1}=\Delta y$. This represents the conditional probability that two bits of information, produced in the same year for a pair of technologies *t* and $t^{\prime }$ reach product *p* after Δy years. We define signal ϕ(Δy) as the fraction of significant $\Lambda_{tt^{\prime }}^{p}\left(\Delta y\right)$ (at the α=0.01 significance level, according to the probability distribution of the null model) for combinations of *t*, $t^{\prime }$, and *p* chosen for selected matrix regions. In general, we consider a population of motifs equal to 5500 units (see below).

## 3. Results

**Figure 1:**
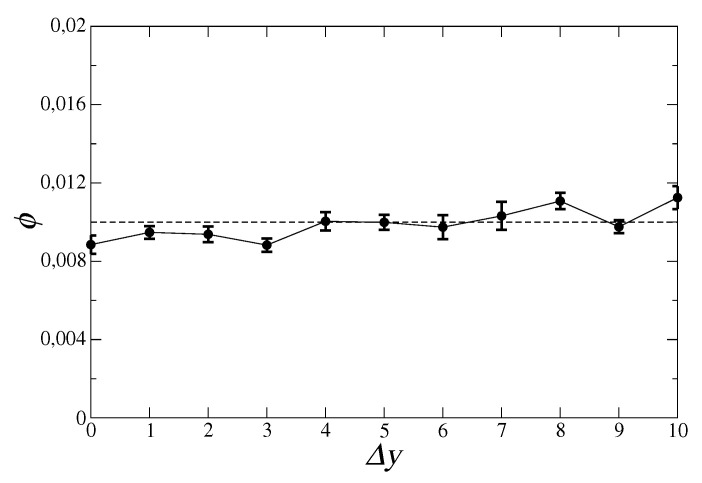
Mean signal ϕ computed over 5500 combinations of *t*, $t^{\prime }$, and *p* chosen at random, for different values of time lag Δy. Error bars represent the standard deviation over year pairs giving the same time lag, whereas the dotted line is significance level α.

As a first test, we report mean signal ϕ, that is, the signal averaged over all combinations of *t*, $t^{\prime }$, and *p*. Since the total number of such motifs is extremely large, we chose 5500 motifs at random and took their mean signal as representative of the global average. Figure 1 shows that ϕ basically remains within one standard deviation from noise level α, indicating that the mean signal within the data is negligible.

**Figure 2:**
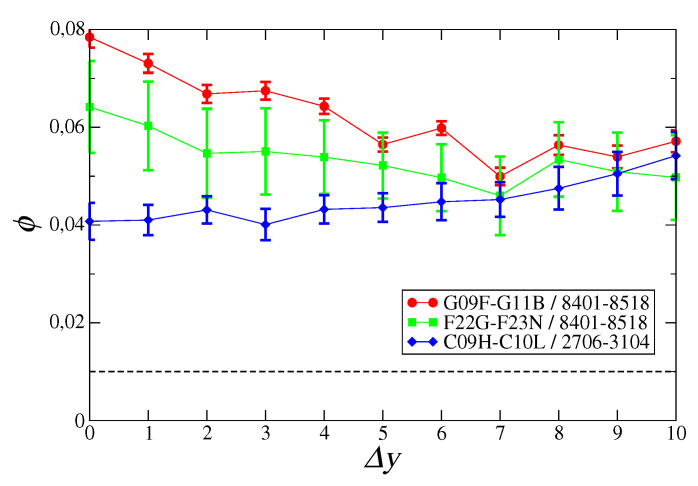
Mean signal ϕ computed over 5500 combinations of *t*, $t^{\prime }$, and *p*, chosen for specific regions of the assist matrix. Red circles: technological codes in the G09F-G11B region—related to sector “physics: instruments of communications, acoustics, optics”, and products in the region 8401–8518 “machinery and metals”. Green squares: technological codes in the F22G-F23N region—related to sector “engineering: various types of machines including steam and combustion”, and products in the 8401–8518 region “machinery and metals”. Blue diamonds: technological codes in the C09H-C10L region—related to sector “chemistry: macromolecular and inorganic compounds, gas and petroleum products”, and products in the 2706–3104 region “inorganic and organic chemicals, pharmaceuticals”. In all cases, error bars represent the standard deviation over the year pairs giving the same time lag, whereas the dotted line is significance level α.

We then report the signal relative to motifs within selected regions of the assist matrix (Figure 2). Specifically, we chose subregions of 11 technologies and 100 products (related to specific technological sectors and product categories), whose total number of Λ motifs was 5500 (since $\Lambda_{tt^{\prime }}^{p}=\Lambda_{t^{\prime }t}^{p}$). From Figure 2 we see that, by selecting coherent sets of technologies and products, the signal is well-enhanced: the presence of a pair of technologies in the capability basket of a country can predict whether that country can successfully export a product, and this happens almost independently on time lag Δy.

**Figure 3:**
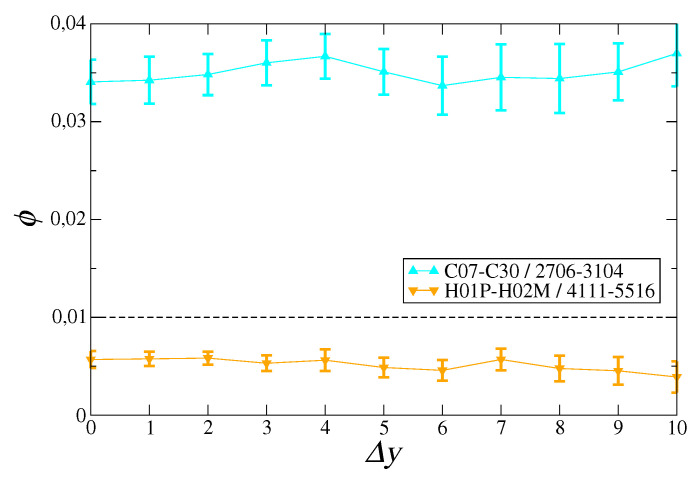
Mean signal ϕ computed over 5500 combinations of *t*, $t^{\prime }$, and *p* chosen for specific regions of the assist matrix. Cyan upper triangles: technological codes in the C07–C30 region related to sector “chemistry” (i.e., a much larger region than that represented in the bottom panel of Figure 2) and products in the 2706–3104 region “inorganic and organic chemicals, pharmaceuticals”. Orange lower triangles: technological codes in the H01P-H02M region—related to sector “physics: electricity”, and products in the 4411–5516 region “textiles”. In all cases, error bars represent the standard deviation over the year pairs giving the same time lag, whereas the dotted line is significance level α.

As consistency checks, we made two exercises, both reported in Figure 3. Firstly, we show that the results we have just presented do not depend on the particular resolution used to choose the motifs. Secondly, we show that, for incoherent technologies and products, we indeed get a much lower signal—even lower than the significance level. In this latter case, a significant development of specific technologies corresponds to a low export level for a given product.

We finally provide a few examples of motifs with a high signal (i.e., with a low *p*-value). To do that, since the total number of motifs is extremely high, so that a complete exploration cannot be performed efficient, we considered the motifs made up of link pairs $B_{tp}\left(y_{1},y_{2}\right)$ and $B_{t^{\prime }p}\left(y_{1},y_{2}\right)$ which were independently those with the highest signal. Table 1 reports some instances of such motifs for specific choice Δy=0. Triplets $t,t^{\prime },p$ appearing in the table indeed seem coherent, and confirm that our method can actually extract meaningful information in an unsupervised way.

## 4. Conclusions

In this work, we provide an effectual way of measuring the combined effect of a set of technologies on one product. In particular, we considered lambda motifs, quantifying the paired effect of two technologies together. In the process of finding relevant combinations, we highlight several results.

First of all, we showed how the combination of multiple technologies has a very different role in different industrial and technological sectors. Technologies within the same sector (we showed examples for physics, engineering and chemistry) tend to show synergies between them in enhancing the chances of successful exports of a product (Figure 2), whereas, looking at two generic technologies there is no such effect (Figure 1). This heterogeneity, while expected, is quantitatively measured here. Secondly, we confirm that co-occurrences between technological activities in a country allow extracting information on shared capabilities, which in turn can inform policymakers and stakeholders of relevant synergies, like those highlighted in Table 1, for specific export markets.

The mapping provided by our approach for the effects of pairs of technologies on products can represent the fundamental building block for the formulation of a powerful instrument to inform policies and industrial strategies about technology transfer. This operational step will be an important aspect of future research.

## Figures and Tables

**Table 1:** Examples of highly significant Λ motifs. *p*-value is averaged over all year pairs $y_{1}=y_{2}$ giving Δy=0. To make this selection, we picked the most significant pairs (individual links) (t,p), and then chose $t^{\prime }$ within the region where the average *p*-value was highest.

*p*-Value	*p*, *t*, $t^{\prime }$
	4701: Wood pulp.
$2\cdot 10^{-4}$	C05B: Lime; magnesia; slag; cements.
	C09K: Materials for applications not otherwise provided for.
	2605: Mineral products.
$5\cdot 10^{-4}$	C21D: Modifying the physical structure of ferrous metals.
	F04F: Pumping of fluid by direct contact of another fluid or by using inertia of fluid to be pumped.
	2605: Mineral products.
$5\cdot 10^{-4}$	C21D: Modifying the physical structure of ferrous metals.
	F04F: Working metallic powder.
	8443: Printing machine.
$5\cdot 10^{-4}$	D02H: Mechanical methods or apparatus in the manufacture of artificial filaments.
	G01T Measurement of nuclear or x-radiation.
	4703: Chemical wood pulp.
$7\cdot 10^{-4}$	D21F: Decorating textiles
	B27C: Planing, drilling, milling, turning, or universal machines.
	2605: Mineral products.
$8\cdot 10^{-4}$	C21D: Modifying the physical structure of ferrous metals.
	FF15B: Systems acting by means of fluids in general.
	4703: Chemical wood pulp.
$2\cdot 10^{-3}$	D21F: Paper-making machines.
	F03D: Wind motors.
	4703: Chemical wood pulp.
$2\cdot 10^{-3}$	D21F: Paper-making machines.
	D06Q: Decorating textiles.
	8519: Sound recording or reproducing apparatus.
$3\cdot 10^{-3}$	G10K: Sound-producing devices.
	G01T: Capacitors, rectifiers, detectors, switching devices.
	8519: Sound recording or reproducing apparatus.
$3\cdot 10^{-3}$	G10K: Sound-producing devices.
	G04f: Time-interval measuring.

## Appendix A. Bipartite Configuration Model (BiCM) and Assist-Matrix Null Model

In order to assess the statistical significance of elements of the assist matrices, we resorted to a null model for bipartite matrices $M^{C,T}\left(y\right)$ and $M^{C,P}\left(y\right)$, built by randomly reshuffling their elements (i.e., the network links connecting nodes in countries layer C to nodes in technologies layers T and products P, respectively), but preserving the diversification of countries and the ubiquity of different innovation activities (i.e., the degrees of the nodes in both layers of the countries–technologies and countries–products bipartite networks). This means that we randomized the signal coming from the network-connectivity patters beyond that encoded in the node degrees. In order to analytically formulate the null model, avoiding to rely on a conditional uniform graph test [28,29], degree constraints are imposed on average, i.e., in a way formally similar to what happens for the canonical ensemble in statistical mechanics with the constraint on energy [23]. This amounts to set a null hypothesis described by the BiCM [20], which is an extension of the configuration model [24] to bipartite networks.

In the following, we use symbols with the tilde for quantities assessed on null-model configurations, and without the tilde for empirically observed values. From an operational viewpoint, the BiCM null model for any binary biadjacency matrix M, representing a (real) empirical bipartite network with two node layers a∈A and b∈B, is built using two main steps:

1. Through a constrained maximum-entropy approach, we define ensemble Ω of bipartite networks that are maximally random, apart from the ensemble average of the node degrees on both layers of the bipartite network that are constrained to generic fixed values. Such an ensemble is thus an instance of an Exponential Random Binary Graph (ERBG).
2. In order to determine the ERBG that best represents the empirical bipartite network, we use a maximal-likelihood argument showing that the mean values of the node degrees have to be taken equal to the observed ones in the empirical network [30]: ${\langle {\tilde{k}}_{a}\rangle }_{\Omega }=k_{a}$, ∀a∈A and ${\langle {\tilde{k}}_{b}\rangle }_{\Omega }=k_{b}$, ∀b∈B, where we have indicated with *k* the observed degrees in the real network, and with $\tilde{k}$ the degrees in a generic configuration of the null model. We remind that $k_{a}=\sum_{b}M_{ab}$ and $k_{b}=\sum_{a}M_{ab}$, and analogously for "tilded" quantities.

Let us start by introducing the ERBG with fixed mean node degrees, and let $\tilde{M}\in \Omega$ be a network configuration in such an ensemble, and $P(\tilde{M})$ be its occurrence probability. By implementing the prescriptions from information theory and statistical mechanics [23,31], the least biased choice of $P(\tilde{M})$ is the one that maximizes informational entropy

(A1) $$S=-\sum_{\tilde{M}\in \Omega }P\left(\tilde{M}\right)\;\ln P\left(\tilde{M}\right),$$

subject to normalization condition $\sum_{\tilde{M}\in \Omega }P\left(\tilde{M}\right)=1$ plus the constraints:

(A2) $${\langle {\tilde{k}}_{a}\rangle }_{\Omega }=\sum_{\tilde{M}\in \Omega }P\left(\tilde{M}\right)\;{\tilde{k}}_{a}\left(\tilde{M}\right)=k_{a}^{*}\;\forall a\in A,\;{\langle {\tilde{k}}_{b}\rangle }_{\Omega }=\sum_{\tilde{M}\in \Omega }P\left(\tilde{M}\right)\;{\tilde{k}}_{b}\left(\tilde{M}\right)=k_{b}^{*}\;\forall b\in B.$$

where $k_{a}^{*}$ and $k_{b}^{*}$ are arbitrarily fixed values for the mean degrees of nodes belonging to layers *A* and *B*. By defining respective Lagrange multipliers ω, ${\left\{\mu_{a}\right\}}_{a\in A}$, and ${\left\{\nu_{b}\right\}}_{b\in B}$ (one for each node of the bipartite network), the probability distribution of all configurations $\tilde{M}\in \Omega$ that maximizes the entropy, satisfying at the same time all the constraints, is determined by the following variational equation:

(A3) $$\begin{array}{cc} 0= & \frac{\delta }{\delta P(\tilde{M})}\left[S+\omega \left(1-\sum_{\tilde{M}\in \Omega }P\left(\tilde{M}\right)\right)+\right.\\ & \left.+\sum_{a\in A}\;\mu_{a}\left(k_{a}^{*}-\sum_{\tilde{M}\in \Omega }P\left(\tilde{M}\right)\;{\tilde{k}}_{a}\left(\tilde{M}\right)\right)+\sum_{b\in B}\;\nu_{b}\left(k_{b}^{*}-\sum_{\tilde{M}\in \Omega }P\left(\tilde{M}\right)\;{\tilde{k}}_{b}\left(\tilde{M}\right)\right)\right]. \end{array}$$

It is a matter of simple algebra to show that the solution of this equation is:

(A4) $$P\left(\tilde{M}\;|\;\left\{\mu_{a}\right\},\left\{\nu_{b}\right\}\right)=e^{-H(\tilde{M}\;|\;\left\{\mu_{a}\right\},\left\{\nu_{b}\right\})}/Z\left(\left\{\mu_{a}\right\},\left\{\nu_{b}\right\}\right)\;,$$

where function $H(\tilde{M}\;|\;\left\{\mu_{a}\right\},\left\{\nu_{b}\right\})$ is usually called the Hamiltonian of graph configurations

(A5) $$H\left(\tilde{M}\;|\;\left\{\mu_{a}\right\},\left\{\nu_{b}\right\}\right)=\sum_{a\in A}\mu_{a}\;{\tilde{k}}_{a}\left(\tilde{M}\right)+\sum_{b\in B}\nu_{b}\;{\tilde{k}}_{b}\left(\tilde{M}\right),$$

and $Z(\left\{\mu_{a}\right\},\left\{\nu_{b}\right\})$ is the corresponding partition function

(A6) $$Z\left(\left\{\mu_{a}\right\},\left\{\nu_{b}\right\}\right)=e^{\omega +1}=\sum_{\tilde{M}\in \Omega }e^{-H(\tilde{M}\;|\;\left\{\mu_{a}\right\},\left\{\nu_{b}\right\})}.$$

Equations (A4)–(A6) above define the network ensemble known as the BiCM model.

Note that, as we have implemented only local constraints, i.e., the mean node degrees, Equation (A4) can be rewritten as the product of single-link probability distributions over all pair of nodes belonging, respectively, to the two different layers [20]:

(A7) $$P\left(\tilde{M}\;|\;\left\{\mu_{a}\right\},\left\{\nu_{b}\right\}\right)=\prod_{a\in A}\prod_{b\in B}\pi_{ab}^{{\tilde{M}}_{ab}}\;{\left(1-\pi_{ab}\right)}^{{\tilde{M}}_{ab}},$$

where $\pi_{ab}$ is simply the probability of the link between nodes a∈A and b∈B:

(A8) $$\pi_{ab}={\langle {\tilde{M}}_{ab}\rangle }_{\Omega }=\sum_{\tilde{M}\in \Omega }{\tilde{M}}_{ab}\;P\left(\tilde{M}\;|\;\left\{\mu_{a}\right\},\left\{\nu_{b}\right\}\right)=\frac{\eta_{a}\;\theta_{b}}{1+\eta_{a}\;\theta_{b}}$$

with $\eta_{a}=e^{-\mu_{a}}$ and $\theta_{b}=e^{-\nu_{b}}$. In other words, the existence of each link is an independent event whose probability is function only of the Lagrange multipliers associated to the node pairs defining the links. The values of the Lagrange multipliers are determined by the constraints of Equation (A2), which can be rewritten in terms of the derivatives of the partition function:

(A9) $$-\frac{\partial }{\partial \mu_{a}}\ln Z\left(\left\{\mu_{a}\right\},\left\{\nu_{b}\right\}\right)\equiv {\langle {\tilde{k}}_{a}\rangle }_{\Omega }=k_{a}^{*}\;\forall a\in A,\;-\frac{\partial }{\partial \nu_{b}}\ln Z\left(\left\{\mu_{a}\right\},\left\{\nu_{b}\right\}\right)\equiv {\langle {\tilde{k}}_{b}\rangle }_{\Omega }=k_{a}^{*}\;\forall b\in B.$$

Equations (A4)–(A8) define the generic EBRG with fixed mean degrees of nodes on both layers of the bipartite network.

Once the generic EBRG has been defined, we can move to the step of determining, among all possible EBRGs, the optimal null model for a given *real* biadjacency matrix (i.e., a bipartite network) M. Equivalently, we have to choose the best values for ${\left\{k_{a}^{*}\right\}}_{a\in A}$ and ${\left\{k_{b}^{*}\right\}}_{b\in B}$, i.e., for Lagrange multipliers ${\left\{\mu_{a}\right\}}_{a\in A}$ and ${\left\{\nu_{b}\right\}}_{b\in B}$, in relation to the connectivity properties of M.

To this aim, we write the log-likelihood function [30]

(A10) $$L\left(\left\{\mu_{a}\right\},\left\{\nu_{b}\right\}\right)=\ln P\left(M\;|\;\left\{\mu_{a}\right\},\left\{\nu_{b}\right\}\right)=\sum_{a\in A}k_{a}\;\ln\eta_{a}+\sum_{b\in B}k_{b}\;\ln\theta_{b}-\sum_{a\in A}\sum_{b\in B}\ln\left(1+\eta_{a}\;\theta_{b}\right),$$

where $P(M\;|\;\left\{\mu_{c}\right\},\left\{\nu_{a}\right\})$ is the probability measure of Equation (A7) evaluated for a configuration coinciding with real network M, while ${\left\{k_{a}\right\}}_{a\in A}$ and ${\left\{k_{b}\right\}}_{b\in B}$ are the node degrees of M. The best values for ${\left\{\eta_{a}\right\}}_{a\in A}$ and ${\left\{\theta_{b}\right\}}_{b\in B}$ (or equivalently ${\left\{\mu_{a}\right\}}_{a\in A}$ and ${\left\{\nu_{b}\right\}}_{b\in B}$) are therefore obtained by maximizing such log likelihood in these parameters. It is simple to show that this amounts to solve the system of ∥A∥+∥B∥ equations in ∥A∥+∥B∥ unknowns (where ∥A∥ and ∥B∥ simply indicate the number of nodes, respectively, in the two layers, *A* and *B*, of M):

(A11) $$\left\{\begin{array}{c} \sum_{b\in B}\frac{\eta_{a}\;\theta_{b}}{1+\eta_{a}\;\theta_{b}}=k_{a}\;\forall a\in A\\ \sum_{a\in A}\frac{\eta_{a}\;\theta_{b}}{1+\eta_{a}\;\theta_{b}}=k_{b}\;\forall b\in B \end{array}\right.$$

which exactly amounts to choosing $k_{a}^{*}=k_{a}\forall a\in A$ and $k_{b}^{*}=k_{b}\forall b\in B$. Finally, the null model for a real bipartite network M is defined by Equations (A7) and (A8), with the Lagrange multipliers set by Equation (A11). This recipe can therefore be applied to construct an appropriate null model for all empirical bipartite networks ${\left\{M^{C,T}\left(y\right),M^{C,P}\left(y\right)\right\}}_{y_{min}\leq y\leq y_{max}}$ obtained, respectively, by PATSTAT and COMTRADE data.

In order to build a null model for assist matrices $B^{T\to P}\left(y_{1},y_{2}\right)$, and consequently for the Λ motifs defined by Equation (4), we have to now compose the null models for bipartite networks $M^{C,T}\left(y_{1}\right)$ and $M^{C,P}\left(y_{2}\right)$. This is done by contracting the two BiCMs for matrices $M^{C,T}\left(y_{1}\right)$ and $M^{C,P}\left(y_{2}\right)$ along the country dimension, as for Equation (3). We have:

(A12) $${\tilde{B}}_{tp}\left(y_{1},y_{2}\right)=\sum_{c=1}^{N_{c}}\frac{{\tilde{M}}_{ct}\left(y_{1}\right)}{{\tilde{k}}_{t}\left(y_{1}\right)}\frac{{\tilde{M}}_{cp}\left(y_{2}\right)}{{\tilde{k}}_{c}^{\left(p\right)}\left(y_{2}\right)}\;,$$

where ${\tilde{k}}_{t}\left(y_{1}\right)=\sum_{c}{\tilde{M}}_{ct}\left(y_{1}\right)$ and ${\tilde{k}}_{c}^{\left(p\right)}\left(y_{2}\right)=\sum_{p}{\tilde{M}}_{cp}\left(y_{2}\right)$ are respectively the ubiquity of technology *t* and the product diversification of country *c* in the two single configurations for the BiCM null models for the two bipartite networks countries-technologies of year $y_{1}$ and countries-products of year $y_{2}$. In other words, starting from the two BiCM ensembles for $M^{C,T}\left(y_{1}\right)$ and $M^{C,P}\left(y_{2}\right)$ we build by composition an ensemble of configurations of bipartite networks $\Omega^{T\to P}\left(y_{1},y_{2}\right)$ with link weights given by Equation (A12). The probability distributions of elements ${\tilde{B}}_{tp}\left(y_{1},y_{2}\right)$, describing the null model, can be, in principle, obtained using exact techniques [26,27]. However, due to the strong non-Gaussianity of such distributions, we adopt a more practical sampling technique: starting from the BiCMs for $M^{C,T}\left(y_{1}\right)$ and $M^{C,P}\left(y_{2}\right)$, we use Equations (A7), (A8) and (A12) to generate null assist matrices and populate related ensemble $\Omega^{T\to P}\left(y_{1},y_{2}\right)$ to estimate the full probability distributions. In a similar way, by using composition Equation (4) for ensemble $\Omega^{T\to P}\left(y_{1},y_{2}\right)$

(A13) $${\tilde{\Lambda }}_{tt^{\prime }}^{p}\left(y_{1},y_{2}\right)={\tilde{B}}_{tp}\left(y_{1},y_{2}\right){\tilde{B}}_{t^{\prime }p}\left(y_{1},y_{2}\right)$$

and averaging over all pairs of years $y_{1}$ and $y_{2}$ with fixed delay $\Delta y=y_{2}-y_{1}$ to obtain ${\tilde{\Lambda }}_{tp}\left(\Delta y\right)$, we can construct the null distribution of Λ motifs for each triple $t,t^{\prime },p$ and delay Δy.

The generic observed element $\Lambda_{tt^{\prime }}^{p}\left(\Delta y\right)$ is then considered statistically significant depending on the *p*-value that we can infer from its distribution under the null hypothesis. The specific threshold for statistical significance and the size of the generated ensemble vary on the performed exercises (as highlighted in the text). In our case, we fixed the statistical significance level at α=0.01. It is useful to recall that the two choices, the threshold and the size of the ensemble, are not unrelated: the higher the threshold we want to test, the bigger the sample we require. We consequently extracted, for each couple of years $y_{1}$ and $y_{2}$, two ensembles of 1000 configurations/matrices for two BiCM null models ${\tilde{M}}^{C,T}\left(y_{1}\right)$ and ${\tilde{M}}^{C,P}\left(y_{2}\right)$; by one-to-one contracting the configurations in the two ensembles, we obtain 1000 values of ${\tilde{\Lambda }}_{tt^{\prime }}^{p}\left(y_{1},y_{2}\right)$ to finally determine the statistical significance of observed value $\Lambda_{tt^{\prime }}^{p}\left(\Delta y\right)$, as explained in Section 2.

A final comment on the issue of multiple hypothesis testing is in order here. Since we perform many statistical tests at once (one for each motif of the assist matrices), in order to determine the true significant elements, we should use a correction for the significant threshold (such as Bonferroni or False Discovery Rate). However, signal ϕ(Δy) is meant to measure the average outcome of a statistical test over all possible motifs in the network, and as such we do not need to implement any correction for the significance threshold.
